# The Knowledge, Attitudes, and Practices of Primary Healthcare Physicians in the Al Qassim Region, Saudi Arabia Regarding Travel Medicine: A Cross-Sectional Study

**DOI:** 10.7759/cureus.52772

**Published:** 2024-01-23

**Authors:** Ebtehal S Almogbel, Shujaa M Almutairi, Ahmed S Almuzaini, Abdulwahab A Alduraibi, Abdulaziz Almutairi, Abdulmalik S Almarshad, Mosaid Altwaijri, Sultan Alharbi

**Affiliations:** 1 Department of Family and Community Medicine, College of Medicine, Qassim University, Buraydah, SAU; 2 College of Medicine, Qassim University, Buraydah, SAU

**Keywords:** practice, attitude, knowledge, saudi arabia, tourist, vision 2030, primary care physician, travel medicine

## Abstract

Background and objective

In the age of globalization, diseases associated with travel have emerged as a focal point of public health interest. This has become particularly relevant in Saudi Arabia after the changes in tourism policy in recent years. Primary care physicians are expected to suspect diseases of importance in certain geographic areas. They should dispense pre- and post-travel advice. This study aimed to assess the knowledge, attitudes, and practices of primary care physicians in the Al Qassim region, Saudi Arabia regarding travel medicine.

Methods

This cross-sectional study was conducted in the Al Qassim region, Saudi Arabia between October and November 2023. We reached out to all primary care physicians in the region regardless of their gender, nationality, and years of experience. The data were collected using a self-administered questionnaire, which was designed based on the available literature and validated by experts.

Results

A total of 197 physicians participated in the study; 74% were male, 46% were general practitioners, and 48% had 5-10 years of experience. More than half (51%) of the participants reported a weekly patient load of 50-100, while 47% engaged with 5-10 travelers annually; 53% provided travel health advice and a quarter of primary healthcare physicians never attended travel update sessions or conferences. In the last six months, 48% and 43% of the physicians conducted pre- and post-travel consultations respectively. Approximately 49.2% demonstrated a fair knowledge of the topic. Factors associated with fair knowledge included non-Saudi nationality, age below 30 years, minimal traveler exposure, and infrequent conference attendance (p<0.05). A positive attitude was linked to being under 30 years old, having <5 years of experience, seeing <5 travelers yearly, and possessing a fair knowledge of the topic (p<0.05).

Conclusions

Overall, about half of the physicians in the Al Qassim region engage with travelers and demonstrate good attitudes and practices toward travel medicine. Opening Saudi borders to tourism necessitates the inclusion of travel medicine in continuing medical education programs to prepare primary care physicians to care for travelers more efficiently.

## Introduction

The ease of travel in the 20th century was associated with an increase in imported infections and the emergence of drug resistance worldwide [1]. This led to the development of the field of travel medicine and resulted in the founding of the International Society of Travel Medicine (ISTM) in 1991 [2]. This is a dynamic field of specialization with a focus on pre-travel preventative care as well as during-travel and post-travel illnesses [3]. It encompasses vaccine-preventable diseases, avoiding insects, malaria, diarrhea, and infectious diseases prophylaxis, self-treatment of traveler's diarrhea, sexual health, promoting responsible behavior, travel medical insurance, and other individualized topics depending on risk assessment [2].

Saudi Arabia is a unique nation because it is home to the holy cities of Mecca and Medina. Almost three million pilgrims gather annually between the 8th and 12th days of the 12th lunar month for Hajj in Saudi Arabia [4]. Around 10 million people visit Saudi Arabia annually for Umrah [5]. Both Hajj and Umrah are Islamic spiritual practices associated with large mass gatherings [6]. Saudi Arabian authorities coordinate issuing of visas from more than 187 countries to ensure travelers are adequately vaccinated and prepared before they embark on their pilgrimage [6]. The Saudi healthcare system is well-equipped to oversee the well-being of pilgrims during their stay and prevent the emergence of disease outbreaks [6]. According to the Saudi Ministry of Tourism, the total number of outbound tourists in 2022 exceeded 16 million [7]. Also, travel is considered an essential part of the lives of Saudi citizens with millions of them traveling outside the country every year [8].

Primary care physicians provide preventive care and are considered the citizens' first point of contact in the healthcare system [9]. Travel medicine is a relatively new discipline with only a few subject specialists practicing it currently. Therefore, it should be integrated within primary care to ensure optimal preventive care and mitigate the risks of travel [9,10]. This study aimed to assess the knowledge, attitudes, and practices of primary care physicians in the Al Qassim region regarding travel medicine.

## Materials and methods

Study setting and inclusion criteria

An observational cross-sectional study was conducted in the Al Qassim region in Saudi Arabia between October and November 2023. The inclusion criteria for this study were as follows: all general practitioners and family medicine specialists working in the Qassim region.

Sample size calculation

The sample size was calculated using the population formula in the Raosoft sample size calculator [11]. The total number of primary care physicians in the Al Qassim region is 320. The required sample size was initially determined to be 175, and taking into account a 10% non-response rate, a final sample size of 193 was agreed upon. The questionnaire was distributed to the physicians on-site and through various social media platforms. A total of 197 responses were received.

Data collection tool

The data were collected by using an electronic self-administered questionnaire and in person. A pilot study involving 20 participants was conducted and participants' feedback was used to improve clarity and understandability. The questionnaire was designed based on the available literature and validated by experts [9,12,13]. It comprised three sections. The first section gathered sociodemographic data. The second section evaluated knowledge about travel medicine through 16 questions about required visits before travel, recommended vaccines and their contraindications, transmission routes of certain infections, and regions known for specific endemic diseases. The second section also assessed attitudes toward travel medicine, barriers, and sources of knowledge. The third section delved into the practice of travel medicine, including the number of pre- and post-travel consultations in the past six months, consultation duration, number of travelers seen annually, and attendance at travel medicine-related events.

Statistical analysis

Data were analyzed using SPSS Statistics v. 27 (IBM Corp., Armonk, NY). Descriptive statistics were employed: mean ±SD for continuous variables and numbers (percentages) for categorical ones. To assess the physicians' knowledge, the correct answers were assigned a score of 2, the wrong ones coded 0, and the unsure answers were coded 1. The total score was calculated, and its normality was assessed using a histogram and Shapiro-Wilk test. Those who scored ≥mean were considered to have a "fair knowledge". The chi-square and Fisher's exact tests were used to determine the predictors of knowledge and attitudes of the participants. A p-value of less than 0.05 was considered statistically significant.

Ethical considerations

The ethical approval was obtained from the regional research ethics committee of Qassim region, Kingdom of Saudi Arabia (IRB No: 607-44-14807). Participation in the study was voluntary and every participant had the right to withdraw at any time without inviting any penalty. Informed consent was obtained from all the participants. Their privacy and confidentiality were ensured. There were no risks or any form of harm from participation in the study.

## Results

A total of 197 physicians participated in the study: 146 (74%) were male, 156 (79%) were Saudi, and 74 (38%) were aged 30-39 years. Approximately 91 (46%) were general practitioners and 94 (48%) had 5-10 years of experience (Table 1).

**Table 1:** Demographic characteristics of the participants (N=197)

Characteristic	N (%)
Age group, years	
<30	71 (36%)
30-39	74 (38%)
40-49	43 (22%)
≥50	9 (4.6%)
Gender	
Female	51 (26%)
Male	146 (74%)
Nationality	
Non-Saudi	41 (21%)
Saudi	156 (79%)
Specialty	
Resident	77 (39.1%)
Senior registrar	17 (8.6%)
General practitioner	91 (46%)
Consultant	12 (6.1%)
Clinical experience, years	
<5	65 (33%)
5-10	94 (48%)
11-15	28 (14%)
>15	10 (5.1%)

Of the participants, 100 (51%) reported meeting 50-100 patients per week and 58 (47%) met 5-10 travelers per year. More than half (n=104, 53%) of respondents stated that they give health advice to travelers, with 60 (48%) spending 5-10 minutes for consultation. Of note, 47 (24%) participants never attended travel medicine updates or conferences. In the last six months, 95 (48%) and 85 (43%) of participants conducted pre- and post-travel consultations respectively (Table 2).

**Table 2:** Practice of travel medicine among primary healthcare physicians (N=197)

Characteristic	N (%)
Number of patients seen per week	
<50	45 (23%)
50-100	100 (51%)
101-150	38 (19%)
>150	14 (7.1%)
Do you give health advice to travelers?	
Yes	104 (53%)
No	93 (47%)
Number of travelers seen per year	
<5	26 (21%)
5-10	58 (47%)
11-20	31 (25%)
>20	8 (6.5%)
Unknown	74
Duration of health advice to travelers, minutes	
<5	32 (26%)
5-10	60 (48%)
11-20	30 (24%)
>20	3 (2.4%)
Unknown	72
Do you attend travel medicine updates or conferences?	
Always	23 (12%)
Sometimes	65 (33%)
Rarely	62 (31%)
Never	47 (24%)
Have you given pre-travel consultation in the past 6 months?	
Yes	95 (48%)
No	102 (52%)
Have you given post-travel consultation during the past 6 months?	
Yes	85 (43%)
No	112 (57%)

The age group of <30 years, seeing less than 5-10 travelers per year, and spending less than five minutes giving health advice were associated with having a high level of knowledge of the topic (p<0.05). Having more than 15 years of experience was linked to possessing fair knowledge. Those who rarely attended travel medicine updates or conferences had higher levels of knowledge than those who always attended them (p<0.001) (Table 3).

**Table 3:** Determinants of travel medicine-related knowledge among primary healthcare physicians *Pearson's Chi-squared test; p-value significant at <0.05 GP: general practitioner

Characteristic	No fair knowledge, n (%) (n=100)	Fair knowledge, n (%) (n=100)	P-value*
Gender			0.5
Female	24 (24%)	27 (28%)	
Male	76 (76%)	70 (72%)	
Age group, years			0.016
<30	27 (27%)	44 (45%)	
30-39	38 (38%)	36 (37%)	
40-49	29 (29%)	14 (14%)	
≥50	6 (6.0%)	3 (3.1%)	
Specialty			0.9
Resident	41 (41%)	36 (37%)	
Senior registrar	8 (8.0%)	9 (9.3%)	
GP	44 (44%)	47 (48%)	
Consultant	7 (7.0%)	5 (5.2%)	
Clinical experience, years			<0.001
<5	14 (14%)	51 (53%)	
5-10	67 (67%)	27 (28%)	
11-20	17 (17%)	11 (11%)	
>15	2 (2.0%)	8 (8.2%)	
Number of patients seen per week			0.4
<50	20 (20%)	25 (26%)	
50-100	55 (55%)	45 (46%)	
101-150	20 (20%)	18 (19%)	
>150	5 (5.0%)	9 (9.3%)	
Do you give health advice to travelers?	55 (55%)	49 (51%)	0.5
Number of travelers seen per year			0.002
<5	9 (13%)	17 (30%)	
5-10	28 (42%)	30 (54%)	
11-20	25 (37%)	6 (11%)	
>20	5 (7.5%)	3 (5.4%)	
Unknown	33	41	
Duration of health advice to travelers, minutes			<0.001
<5	11 (16%)	21 (38%)	
5-10	31 (45%)	29 (52%)	
11-20	24 (35%)	6 (11%)	
>20	3 (4.3%)	0 (0%)	
Unknown	31	41	
Do you attend travel medicine updates or conferences?			<0.001
Never	13 (13%)	34 (35%)	
Rarely	27 (27%)	35 (36%)	
Sometimes	43 (43%)	22 (23%)	
Always	17 (17%)	6 (6.2%)	

Ninety-three (47%) participants stated that there is no significance in learning about travel medicine as it is an uncommon field. Although 116 (59%) stated that they did not have time to learn about travel medicine, 165 (84%) acknowledged the importance and usefulness of learning travel medicine. Websites, the Saudi Ministry of Health, and books/journals were the major sources of information regarding travel medicine as cited by 85 (43%), 82 (42%), and 81 (41%) participants respectively (Table 4).

**Table 4:** Attitudes toward travel medicine (N=197) *Multiple-answer question CDC: Centers for Disease Control and Prevention; MOH: Ministry of Health

Characteristic	N (%)
I can’t see the point in learning travel medicine because it is not common in our practice	
No	104 (53%)
Yes	93 (47%)
I haven’t got the time to learn about travel medicine	
No	81 (41%)
Yes	116 (59%)
Learning travel medicine is important, interesting, and useful in our practice	
No	32 (16%)
Yes	165 (84%)
Source of information about travel medicine*	
Websites	85 (43%)
MOH	82 (42%)
Books/journals	81 (41%)
CDC	63 (32%)
Experts/senior staff	59 (30%

Being in the age group of less than 30 years, having less than five years of experience, seeing less than five travelers a year, and having fair knowledge of travel medicine were associated with a positive attitude towards learning travel medicine (p<0.05) (Table 5).

**Table 5:** Determinants of attitudes towards travel medicine *Pearson's Chi-squared test, Fisher's exact test; p-value significant at <0.05 GP: general practitioner

Characteristic	Learning travel medicine is important, interesting, and useful		
	No, n (%) (n=32)	Yes, n (%) (n=165)	P-value*
Gender			>0.9
Female	8 (25%)	43 (26%)	
Male	24 (75%)	122 (74%)	
Nationality			0.082
Non-Saudi	3 (9.4%)	38 (23%)	
Saudi	29 (91%)	127 (77%)	
Age group, years			<0.001
<30	4 (13%)	67 (41%)	
30-39	10 (31%)	64 (39%)	
40-49	14 (44%)	29 (18%)	
≥50	4 (13%)	5 (3.0%)	
Specialty			0.3
Resident	10 (31%)	67 (41%)	
Senior registrar	3 (9.4%)	14 (8.5%)	
GP	15 (47%)	76 (46%)	
Consultant	4 (13%)	8 (4.8%)	
Clinical experience, years			0.016
<5	4 (13%)	61 (37%)	
5-10	19 (59%)	75 (45%)	
11-15	8 (25%)	20 (12%)	
>15	1 (3.1%)	9 (5.5%)	
Number of patients seen per week			0.2
<50	6 (19%)	39 (24%)	
50-100	13 (41%)	87 (53%)	
101-150	11 (34%)	27 (16%)	
>150	2 (6.3%)	12 (7.3%)	
Do you give health advice to travelers?			0.2
No	12 (38%)	81 (49%)	
Yes	20 (63%)	84 (51%)	
Number of travelers seen per year			<0.001
<5	0 (0%)	26 (26%)	
5-10	8 (33%)	50 (51%)	
11-20	12 (50%)	19 (19%)	
>20	4 (17%)	4 (4.0%)	
Unknown	8	66	
Knowledge level			0.001
Fair knowledge	16 (50%)	128 (78%)	
No fair knowledge	16 (50%)	37 (22%)	

**Figure 1:**
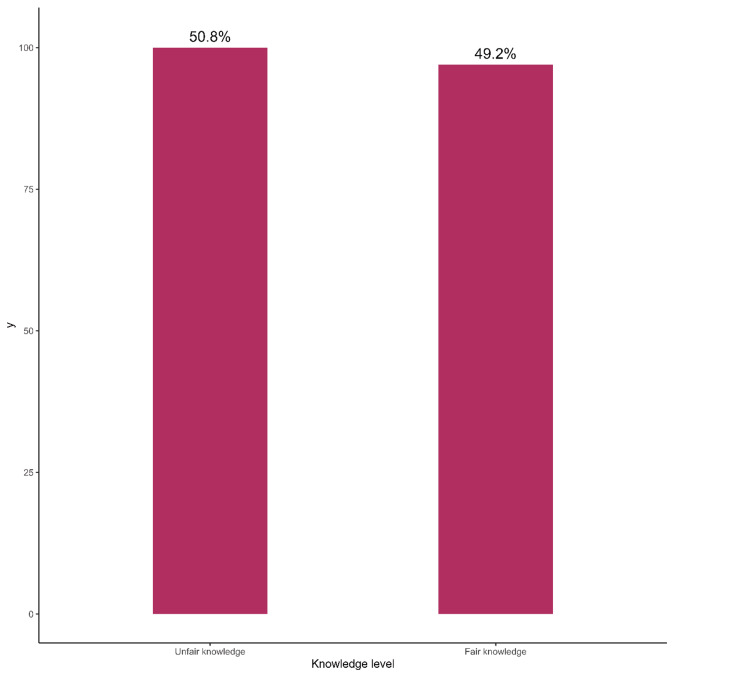
Knowledge of travel medicine among primary healthcare physicians

**Figure 2:**
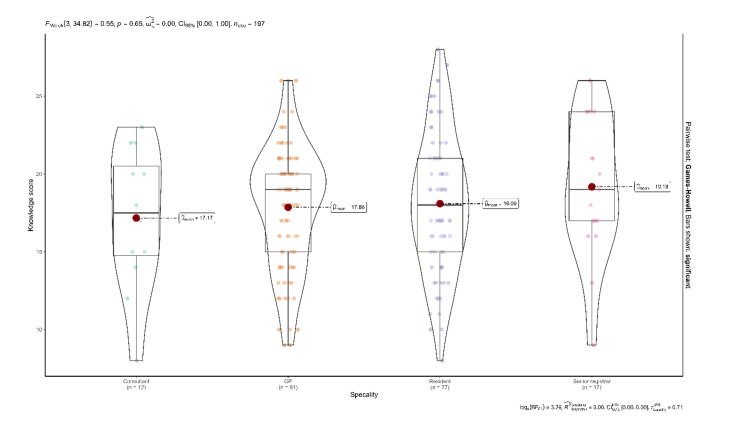
Knowledge of travel medicine among physicians by specialty GP: general practitioner

**Figure 3:**
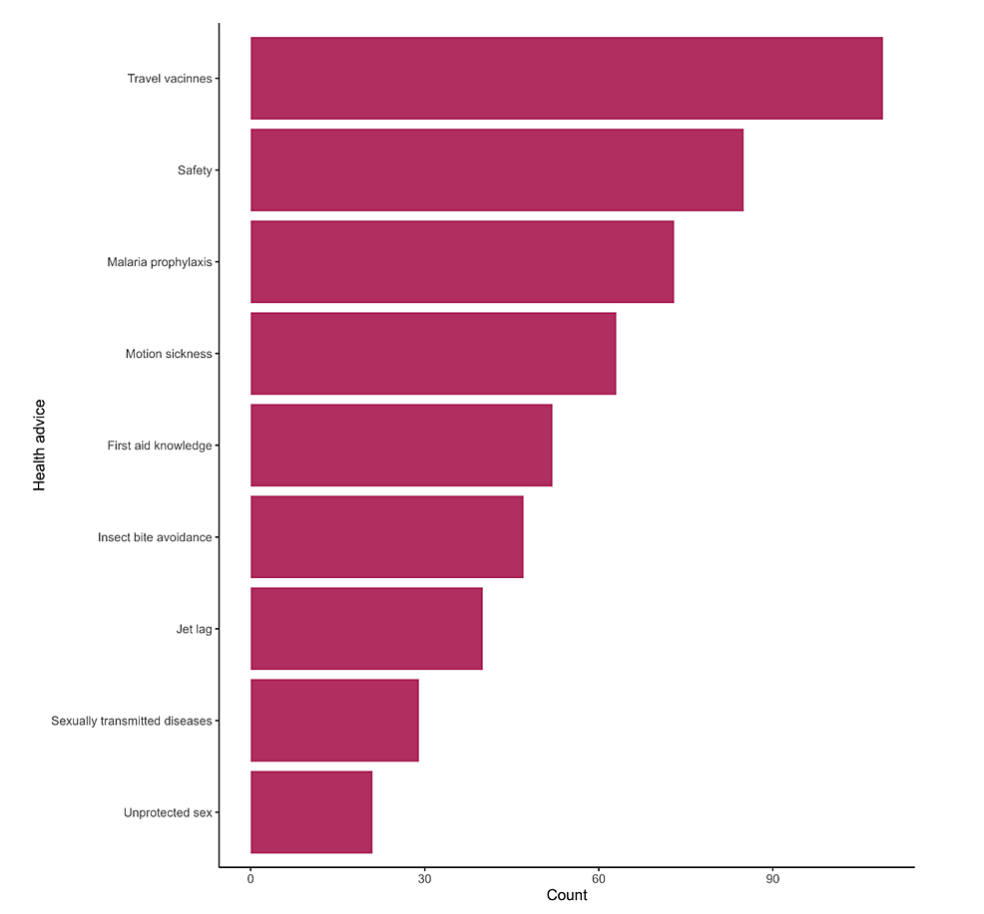
Travel medicine guidance provided by primary healthcare physicians

Among the participants, 49.2% had a fair knowledge of travel medicine (Figure 1). Senior registrars had a higher level of knowledge about travel medicine than the consultants; however, the association didn't reach statistical significance (p=0.65) (Figure 2). Major health advice provided pertained to vaccines, safety, malaria prophylaxis, and motion sickness, as reported by 109 (55%), 85 (43%), 73 (37%), and 63 (32%) of physicians respectively (Figure 3).

## Discussion

As we approach the year 2030, the field of travel medicine should receive the attention of physicians in all regions of Saudi Arabia. This study aimed to assess the knowledge, attitudes, and practices of primary care physicians in the Al Qassim region, an important tourist destination, regarding travel medicine. In this study, more than a quarter of the participants were females. This aligns with the rising trend of female representation in the healthcare sector in Saudi Arabia, which grew from 4.4% in 1981 to 36.4% in 2018 [14,15]. More than a third were younger than 30 years, and nearly half of the participants had 5-10 years of clinical experience. According to The General Authority for Statistics, youths aged under 35 years constitute 67.0% of the Saudi population [16].

Our data showed that only half of the surveyed physicians are engaged in consultations related to travel medicine and have provided pre- or post-travel advice in the past six months. Almost half of the participants reported seeing 5-10 travelers per year, with only 6.5% attending to more than 20 travelers per year. A study in the USA reported that two-thirds of primary care physicians attend to fewer than 50 patients per year and 5.3% of physicians see more than 500 travelers per year [12]. A similar study conducted in Qatar stated that almost three-quarters of primary care physicians see less than 10 travelers per month while 8.3% of them see more than 20 per month [17]. The use of categories rather than mean or median complicates the comparison between countries. Still, travelers are commonly seen in primary care clinics, a practice that is especially prominent in the USA, which is a popular destination for travelers [18].

Almost half of the surveyed physicians reported spending 5-10 minutes per consultation on travel medicine. Average consultation length varies between countries and may be as low as 48 seconds in Bangladesh or as long as 22.5 minutes in Sweden and is likely to affect the quality of medical care [19]. We cannot ascertain if 5-10 minutes is sufficient for consultation when addressing diseases acquired during travel. Nonetheless, the Infectious Diseases Society of America has stated that pre-travel consultations should take 15-45 minutes and even this will not be enough to address all possible health issues [2]. A possible cause of the short consultation time is the large number of travelers and the significant patient load in PHC centers, but the exact cause should be investigated in future studies. Almost half of the surveyed physicians never or rarely attended a travel medicine update session or conference. This represents a slight improvement compared to a previous study, which reported that nearly three-quarters of physicians never attended events related to travel medicine [9]. Despite this improvement, the proportion is still low given the notable increase in travel in the country.

Almost half of the surveyed physicians thought travel medicine is not important in the Saudi context and a little more than half stated they have no time to learn about travel medicine. Considering the ongoing transformation in the Saudi tourism sector, this attitude of insignificance warrants further investigation. Time is frequently reported as a barrier to continuing medical education in general [20,21]. Mandatory online continuing medical education may help address the issue of time constraints [22]. The most frequently reported sources for acquiring knowledge on travel medicine are websites, the Ministry of Health, and books/journals. This aligns with the findings of another study from Saudi Arabia [9]. The most frequently given advice was related to vaccines, insect bite avoidance, and motion sickness.

Almost half of the surveyed physicians had a fair knowledge of travel medicine, a proportion similar to those who practice it. The factors associated with better knowledge, as well as better attitude, include younger age and less than five years of clinical experience. In September 2019, Saudi Arabia received international tourists for the first time [23]. Before that, it had primarily received pilgrims, and the Al Qassim region was not a primary destination for them. It is reasonable to assume that opening the borders encouraged young physicians to explore the field of travel medicine.

This study is a significant addition to the existing body of knowledge on travel medicine in Saudi Arabia. Its main limitation was the use of a convenient sampling approach. Nonetheless, we included more than half of the physicians in the area to ensure a robust level of representation.

## Conclusions

This is the first study to assess primary care physicians’ knowledge, attitudes, and practices regarding travel medicine in the Al Qassim region of Saudi Arabia. The findings showed that only half of the physicians engage with travelers and hold a positive attitude in terms of practice in the field. Young physicians were more knowledgeable in the field, which reflects the changes in the dynamics related to travel medicine in Saudi Arabia. Given the positive association between knowledge and attitude, we recommend incorporating travel medicine in the continuing medical education activities of physicians in the Al Qassim region to prepare them to care for travelers. Given the significant increase in the number of travelers, specialized travel clinics will be of value in the region, but further research and cost-effectiveness analyses need to be conducted before significant steps are implemented in this field.
